# Effect of tRNA Maturase Depletion on Levels and Stabilities of Ribosome Assembly Cofactor and Other mRNAs in Bacillus subtilis

**DOI:** 10.1128/spectrum.05134-22

**Published:** 2023-02-22

**Authors:** Aude Trinquier, Ciarán Condon, Frédérique Braun

**Affiliations:** a CNRS, Université Paris Cité, Expression Génétique Microbienne, Institut de Biologie Physico-Chimique, Paris, France; Centre National de la Recherche Scientifique, Aix-Marseille Université

**Keywords:** RNA degradation, tRNA processing, translation, chloramphenicol, (p)ppGpp

## Abstract

The impact of translation on mRNA stability can be varied, ranging from a protective effect of ribosomes that shield mRNA from RNases to preferentially exposing sites of RNase cleavage. These effects can change depending on whether ribosomes are actively moving along the mRNA or stalled at particular sequences or structures or awaiting charged tRNAs. We recently observed that depleting Bacillus subtilis cells of their tRNA maturation enzymes RNase P and RNase Z led to altered mRNA levels of a number of assembly factors involved in the biogenesis of the 30S ribosomal subunit. Here, we extended this study to other assembly factor and non-assembly factor mRNAs in B. subtilis. We additionally identified multiple transcriptional and translational layers of regulation of the *rimM* operon mRNA that occur in response to the depletion of functional tRNAs.

**IMPORTANCE** The passage of ribosomes across individual mRNAs during translation can have different effects on their degradation, ranging from a protective effect by shielding from ribonucleases to, in some cases, making the mRNA more vulnerable to RNase action. We recently showed that some mRNAs coding for proteins involved in ribosome assembly were highly sensitive to the availability of functional tRNA. Using strains depleted of the major tRNA processing enzymes RNase P and RNase Z, we expanded this observation to a wider set of mRNAs, including some unrelated to ribosome biogenesis. We characterized the impact of tRNA maturase depletion on the *rimM* operon mRNA and show that it is highly complex, with multiple levels of transcriptional and posttranscriptional effects coming into play.

## INTRODUCTION

The steady-state level of any mRNA in the cell is determined by both its rate of transcription by RNA polymerase and its rate of degradation by RNases. These can work together to increase or decrease gene expression at the transcriptional or posttranscriptional levels in response to environmental stimuli or can pull in opposing directions, resulting in little net gain. While most of the enzymes responsible for RNA decay in Bacillus subtilis are now known (1), how these enzymes are impacted by translation is still a relatively open question. The conventional wisdom is that increased translation leads to increased stability due to the masking of RNase cleavage sites by ribosomes. However, we recently identified an endoribonuclease (Rae1) that actually depends on translation to destabilize mRNAs (2, 3), and we present data here suggesting that antibiotics that cause ribosome pausing can both positively and negatively impact mRNA levels, depending on the severity and time of the dose.

Efficient translation depends on an unlimited supply of functional charged tRNAs. tRNAs are almost universally transcribed as precursors in all living organisms, with both 5′ and 3′ extensions that must be removed to generate tRNAs that can be charged and used in translation. In B. subtilis, tRNAs are matured at their 5′ end by the ubiquitous endoribonuclease RNase P, consisting of a catalytic RNA moiety encoded by the *rnpB* gene and a protein subunit encoded by the *rnpA* gene (4). Their 3′ ends are matured either by the endo/exoribonuclease RNase Z or by a number of redundant 3′ exoribonucleases, depending nominally on whether the tRNA gene carries the CCA motif required for aminoacylation (5). RNase Z processes the one-third of B. subtilis tRNAs lacking an encoded CCA motif through stimulation of its endoribonuclease activity about 200-fold by a uracil residue that naturally occurs ≤2 nucleotides (nt) downstream of the so-called discriminator nucleotide (nt 75) of each of these tRNA precursors (6). The enzyme’s 3′-exoribonuclease activity is required to trim back to nt 75 to allow addition of the CCA motif by nucleotidyltransferase (NTase, or CCAse). Both RNase P and Z are essential in B. subtilis, and depletion of either enzyme inhibits cell growth, presumably due to a lack of functional tRNAs for translation.

Translation can also be inhibited by antibiotics that target the ribosome, such as chloramphenicol (Cm), which targets the peptidyltransferase center (PTC) located on the large ribosomal subunit. Although it was once thought that Cm blocked translation randomly, recent ribosome profiling experiments have shown that Cm preferentially causes ribosomes to stall at particular sites, in particular when alanine (Ala) or serine (Ser) residues have just been incorporated into the nascent peptide (7).

We recently showed that depletion of tRNA maturase activity affects ribosome assembly, leading to a specific 30S subunit late assembly defect (8). While this defect was mostly explained by a RelA-dependent accumulation of the stringent response alarmone (p)ppGpp and inhibition of GTP-dependent assembly factor activity, we also observed that the levels of several mRNAs encoding ribosome assembly cofactors were affected. Notably, the steady-state levels of transcripts encoding the GTPases Era and YqeH were upregulated during tRNA maturase depletion, whereas mRNAs encoding the GTPase CpgA and the RNA chaperone RimM were downregulated. Because RNase P is thought to have very few direct mRNA targets, and because RNase Z depletion had comparable effects on the expression of these mRNAs, we considered it unlikely that the effects observed were directly due to RNase P or RNase Z cleavages in each of these mRNAs. We therefore wished to better understand by which mechanism(s) tRNA maturase depletion affected the levels of the cofactor encoding mRNAs. Since the late 30S ribosome assembly defect observed in tRNA maturase depletion strains was very similar to that observed in both E. coli and B. subtilis Δ*rimM* mutants, we put additional focus on exploring the decrease in *rimM* expression under these conditions.

## RESULTS

### tRNA maturase depletion alters assembly factor mRNA levels.

**FIG 1:**
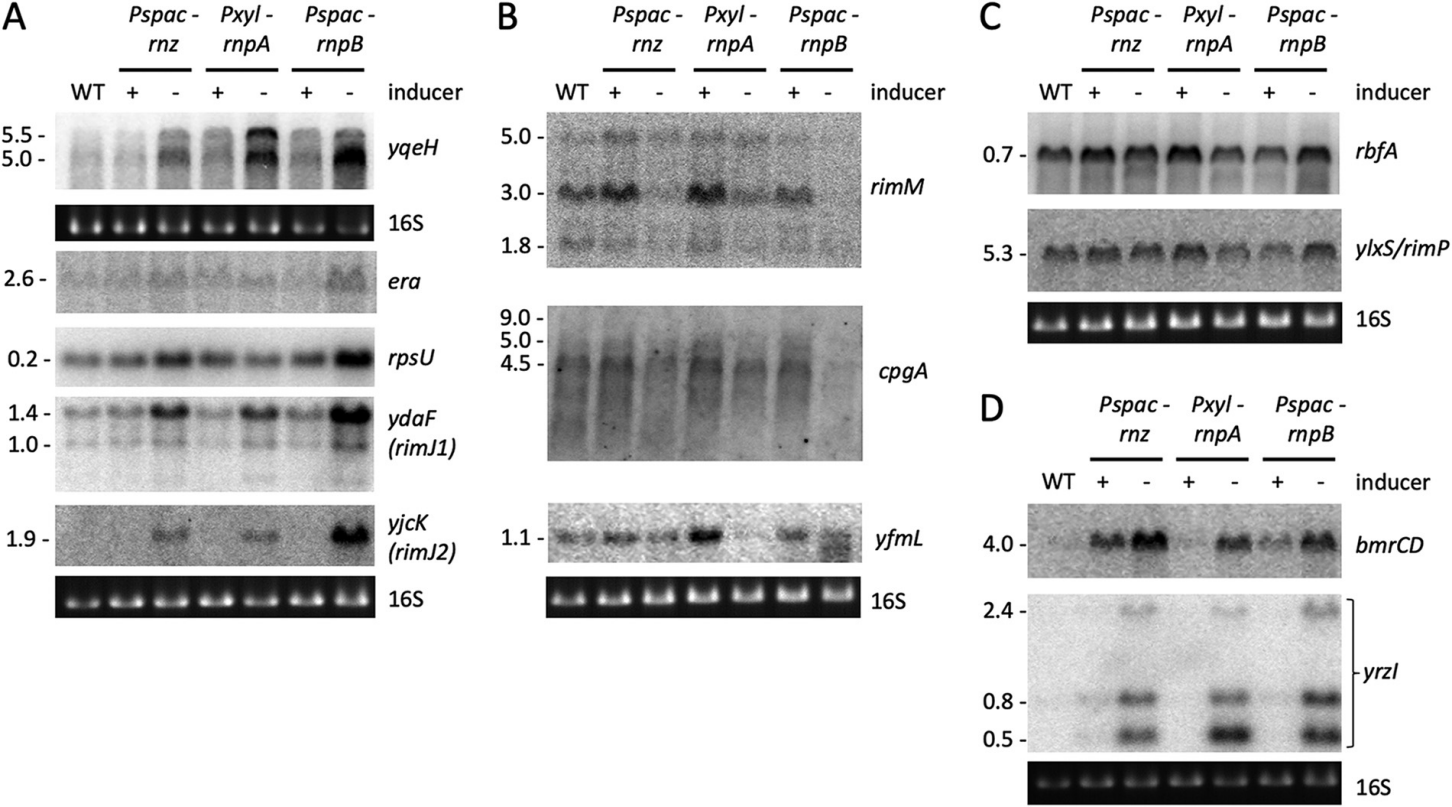
Depletion of tRNA processing enzymes results in perturbed expression of some mRNAs encoding proteins involved in 30S subunit assembly. Northern blots showing (A) up-regulated mRNAs, (B) down-regulated mRNAs, (C) unaffected mRNAs, and (D) mRNAs unrelated to ribosome assembly, present in total mRNA isolated in the presence or absence of inducer as indicated. Note that the basal level of the *bmrCD* transcript, encoding a multidrug transporter, is higher in the *Pspac*-*rnz* and *Pspac*-*rnpB* strains because of the presence of erythromycin in the medium for stable maintenance of the construct. 16S rRNA levels (ethidium bromide stained) are shown as a loading control. Series of blots where a single loading control is shown were stripped and reprobed. The membrane used for panel B is the same as in panel C. The blots for *era*, *yqeH*, *rimM*, and *cpgA* were regenerated as in reference 8 with independent RNA preparations, with permission granted by the publisher for reuse of previously published data. Numbers of repetitions are as follows: *yqeH*, 3; *era*, 3; *rpsU*, 4; *ydaF*, 2; *yjcK*, 2; *rimM*, 3; *cpgA*, 3; *rbfA*, 2; *ylxS*, 2; *yfmL*, 2; *bmrCD*, 2; *yrzI*, 2.

We previously showed that depletion of RNase P or RNase Z results in altered mRNA levels of four key 30S assembly cofactors (Era, YqeH, RimM, and CpgA) (8). The effects of depleting the RNA subunit of RNase P (RnpB) were more severe than those of depleting the protein subunit (RnpA), presumably because the RNA component of RNase P is more rapidly depleted than the protein subunit once transcription is shut off. To ask whether this applied to other mRNAs involved in ribosome biogenesis, we extended this analysis to the expression of several other cofactor and ribosomal protein mRNAs, using xylose (*Pxyl*-*rnpA*) or IPTG (isopropyl-β-d-thiogalactopyranoside)-dependent (*Pspac*-*rnpB* and *Pspac*-*rnz*) promoter constructs to deplete the protein and RNA subunits of RNase P and RNase Z, respectively. The two control transcripts, *yqeH* and *era*, and three new transcripts, *ydaF* and *yjcK* (encoding two potential homologs of the Escherichia coli RimJ acetylase) and *rpsU* (encoding r-protein S21), were globally increased under conditions of tRNA maturase depletion (Fig. 1A), while *rimM* and *cpgA* (controls) and *yfmL* (encoding a DEAD box helicase) transcripts all showed decreased expression, with a visible accumulation of degradation intermediates for *yfmL* (Fig. 1B). Expression of the *rbfA* and *ylxS/rimP* mRNAs was relatively unchanged (Fig. 1C), showing that tRNA maturase depletion does not cause nonspecific perturbation of the expression of all B. subtilis ribosome assembly cofactor genes. Although the primary focus of this study was on assembly factor mRNAs because of the link to a defect in 30S biogenesis, we also asked whether effects of tRNA maturase depletion on other mRNAs could be seen. Indeed, mRNAs from the *yrzI* and *bmrCD* operons, encoding multiple peptides of unknown function and a multidrug resistance pump, respectively, were also upregulated upon RNase P or RNase Z depletion (Fig. 1D), suggesting that this phenomenon is not confined to mRNAs with ribosome-related functions.

### tRNA maturase depletion and the translation inhibitor chloramphenicol alter mRNA stability in a similar manner.

**FIG 2:**
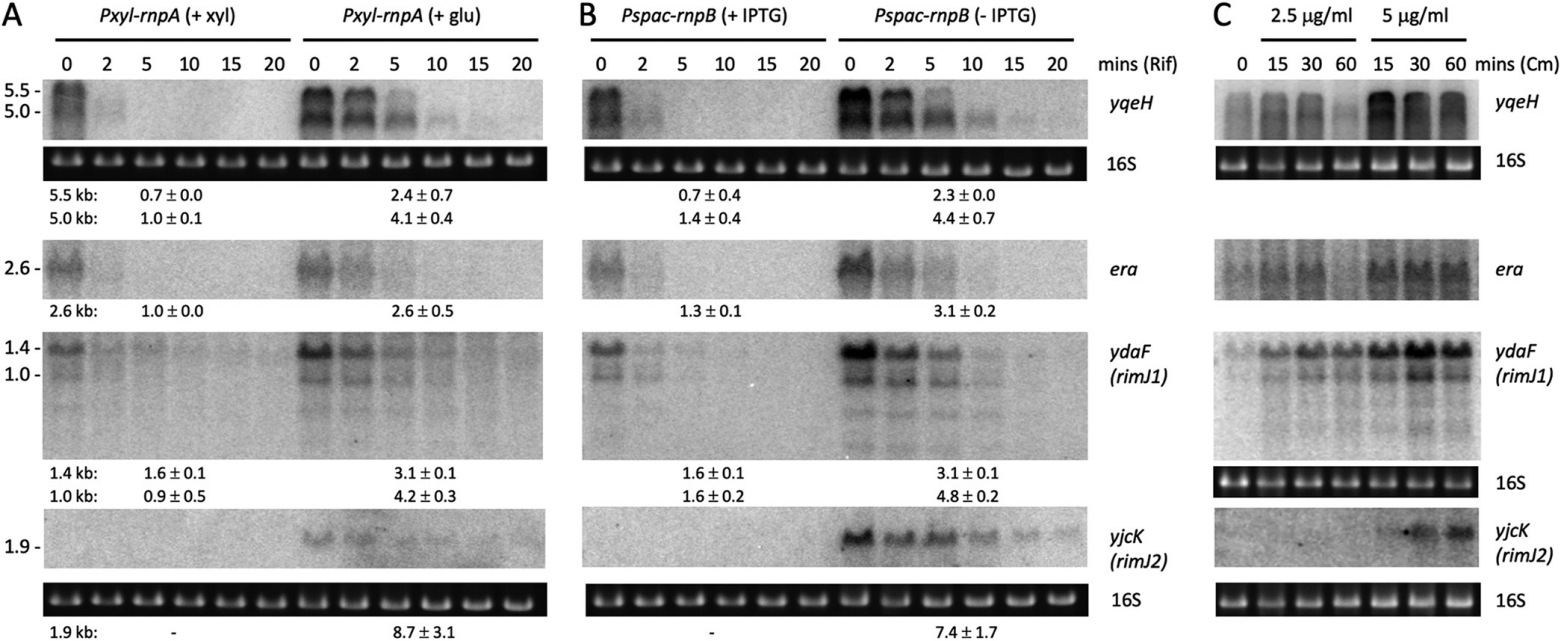
Upregulated mRNAs show increased stability upon tRNA maturase depletion and increased expression levels in the presence of chloramphenicol. Northern blots of total RNA isolated at different times after addition of rifampicin (Rif) to cells grown in the presence or absence of inducer for (A) *rnpA* or (B) *rnpB* expression. Transcript sizes are given in kilobases to the left of the blots, and half-lives are reported under each blot. Note that since *yjcK* gives no signal in the presence of inducer, we cannot rule out a transcriptional effect in this case. (C) Northern blots of total RNA isolated at different times after addition of 0.5× MIC and the MIC of Cm. 16S rRNA levels (ethidium bromide stained) are shown as a loading control. Series of blots where a single loading control is shown were stripped and reprobed. The membrane used to probe *yqeH* is the same as that probed with *yjcK*. Experiments were performed twice, with decay plots and their quantifications given in Fig. S1.

To determine whether tRNA maturase depletion impacted mRNA expression at the transcriptional or posttranscriptional level, we measured the stability of several of these mRNAs after rifampicin treatment in RNase P (RnpA or RnpB)-depleted cultures. The upregulated transcripts (*yqeH*, *era*, *ydaF*, and *yjcK*) were all stabilized during RnpA and RnpB depletion (Fig. 2A and B; also, see Fig. S1 in the supplemental material), suggesting that they are affected by RNase P depletion at the posttranscriptional level. There is evidence that the lack of functional tRNAs can increase ribosome stalling on translated mRNAs (9). Thus, the increased stability of these transcripts could be due to ribosome stalling and the blocking of RNase access to cleavage sites on these mRNAs. To test this hypothesis, we sought to recapitulate the effect by pausing translation in a different manner, using the translation elongation inhibitor Cm. Indeed, the addition of a subinhibitory concentration (2.5 μg/mL) and the MIC (5 μg/mL) of Cm to wild-type (WT) cells also increased the levels of the *yqeH*, *era*, *ydaF*, and *yjcK* mRNAs (Fig. 2C), suggesting that the stabilization of these transcripts in tRNA maturase depletion strains is most likely due to the lack of mature tRNA and ribosome stalling.

**FIG 3:**
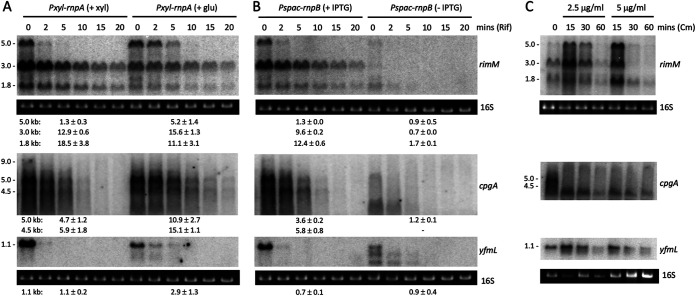
Downregulated mRNAs are subjected to a mixture of transcriptional and posttranscriptional effects upon tRNA maturase depletion and chloramphenicol addition. Northern blots of total RNA isolated at different times after addition of rifampicin (Rif) to cells grown in the presence or absence of inducer for (A) *rnpA* or (B) *rnpB* expression. Transcript sizes are given in kilobases to the left of the blot, and half-lives are reported under each blot. (C) Northern blots of total RNA isolated at different times after addition of 0.5× MIC and the MIC of Cm. 16S rRNA levels (ethidium bromide stained) are shown as a loading control. Series of blots where a single loading control is shown were stripped and reprobed. The membranes used in panel A (top, *yqeH* probe; bottom, *era*, *ydaF*, and *yjcK* probes) are the same as in Fig. 2A (top, *rimM*; bottom, *cpgA* and *yfmL* probes). Similarly, the membranes used in panel B (top and bottom) are the same as in Fig. 2B (top and bottom, respectively). The membrane used to probe *rimH* in panel C is the same as that probed with *era* and *ydaF* in Fig. 2C. Experiments were performed twice, with decay plots and their quantifications given in Fig. S2.

The situation with the downregulated transcripts was more complicated. The major *rimM* (5, 3, and 1.8 kb) and *cpgA* (5 and 4.5 kb) transcripts were strongly destabilized in RNase P RNA subunit (RnpB)-depleted cells (Fig. 3B; Fig. S2), suggesting that the decrease in expression also occurs at a posttranscriptional level in this strain. A similar decrease in expression was seen after 30 min at high Cm concentration (MIC) in WT cells for *rimM* and rapidly upon exposure to Cm for *cpgA* (Fig. 3C), suggesting that this phenomenon is also linked to ribosome stalling. For both *cpgA* and *yfmL*, the major transcripts were processed to shorter forms in the absence of RnpB or in the presence of Cm (Fig. 3B). One possibility is that, in contrast to the upregulated mRNAs, when nonfunctional tRNA precursors accumulate to high levels in the *rnpB* depletion strain, ribosomes eventually stall at sites that preferentially allow RNase access. Another possibility is that the RNAs are largely unoccupied by ribosomes.

In the less severely depleted *rnpA* strain, the full-length (5-kb) *rimM* transcript and the two major *cpgA* mRNAs were stabilized (or showed little effect), rather than being destabilized, as seen for *rnpB* (Fig. 3A). These results suggest that downregulation of *rimM* and *cpgA* arises from a mixture of transcriptional (down) and posttranscriptional (up initially, then down) effects and that one or the other effect predominates depending on the severity of RNase P depletion. Indeed, upon close inspection of Fig. 3C, it can be seen that *rimM* and *cpgA* mRNA levels initially increase at 15 min and then decrease after further exposure to Cm at both subinhibitory and MIC doses. Thus, the Cm effect globally tracks the effect of tRNA depletion, with the weak Cm dose (2.5 μg/mL) mimicking the weak effect of depleting RnpA and the strong Cm dose (5 μg/mL) mimicking the strong effect of depleting RnpB, consistent with the notion of opposing responses to severe versus less severe levels or duration of translation inhibition.

### Identification of *rimM*-containing transcripts sensitive to RNase P depletion.

**FIG 4:**
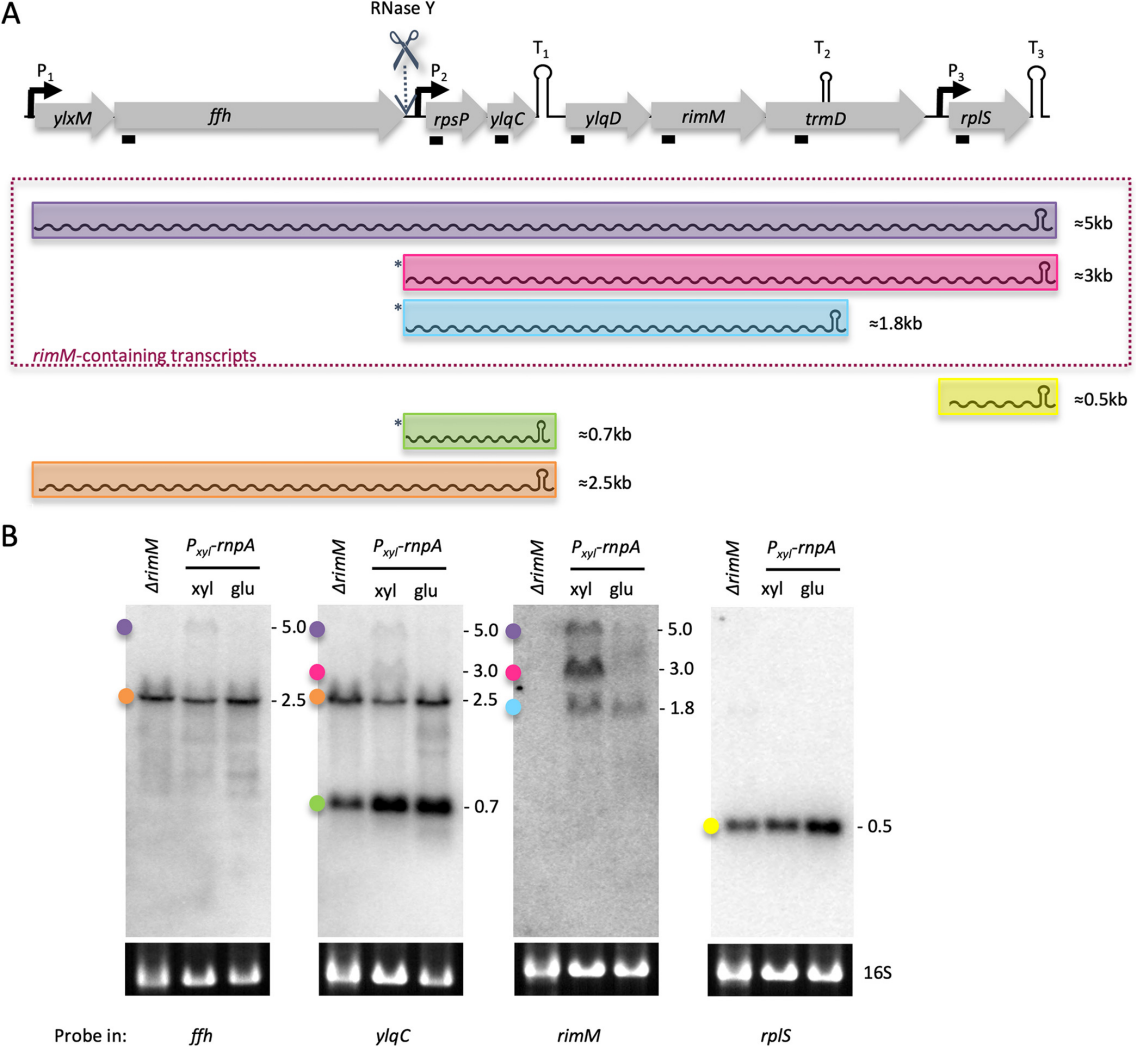
Three of the six transcripts emanating from the *rimM* operon are sensitive to tRNA maturase depletion. (A) Structure of the *rimM* operon. ORFs (not to scale) are shown as gray arrows and transcripts as colored wavy lines. Promoters (P_1_ to P_3_) are represented by black arrows and terminators (T_1_ to T_3_) as hairpins. A known RNase Y cleavage site is indicated. The asterisks indicate transcripts that may be processed by RNase Y but are not distinguishable from P_2_ primary transcripts by Northern blotting. These are designated Y/P_2_ in the text. The black boxes indicate the location of the probe used. (B) Northern blot analysis of total RNA from *Pxyl*-*rnpA* cells grown in the presence or absence of inducer, probed with oligonucleotides targeting different ORFs of the operon (indicated at the bottom). Colored dots correspond to the colors of the transcripts in panel A.

Because the Δ*rimM* phenotype closely fitted the 30S late assembly defect observed in strains depleted of RNase P or RNase Z (8), we attempted to narrow down the determinants of the downregulation of this operon. The *rimM* gene is located in a large operon containing several genes encoding components of the translation machinery: ribosomal protein genes *rpsP* and *rplS* (encoding S16 and L19, respectively), signal recognition particle component genes (*ffh* and *ylxM*), and *trmD* (encoding a tRNA methyltransferase) (Fig. 4A). To identify the gene composition of the three *rimM*-containing transcripts, we performed Northern blots with probes located in open reading frames (ORFs) of the neighboring genes (Fig. S3). In all, six different transcripts originate from this locus (Fig. 4A). Promoters upstream of *ylxM* (P_1_) and *rplS* (P_3_), and terminators downstream of *ylqC* and *rplS* (T_1_ and T_3_, respectively) were identified earlier by transcriptome analysis (10). Our Northern blot analysis suggested that two transcripts originate from P_1_: the full-length mRNA (5 kb) (Fig. 4A, highlighted in purple), which terminates at T_3_, and a shorter transcript (2.5 kb) (Fig. 4A, highlighted in orange) that terminates at T_1_ and does not contain the *rimM* ORF. The smallest species identified (0.5 kb) (Fig. 4A, highlighted in yellow) corresponds to the monocistronic *rplS* transcript (P_3_ to T_3_). Using end enrichment RNA sequencing (Rend-seq), DeLoughery et al. identified a third transcription start site (P_2_) located just upstream of *rpsP* and only 18 nt downstream of an RNase Y cleavage site in the *ffh*-*rpsP* intergenic region, in addition to a potential terminator/attenuator (T_2_) within the *trmD* ORF (11). The three remaining transcripts (0.7, 1.8, and 3 kb [Fig. 4A, highlighted in green, cyan, and pink, respectively, and marked with asterisks]) therefore correspond to either P_2_ primary transcripts or RNase Y-processed transcripts originating from P_1_, which terminate at T_1_, T_2_, and T_3_, respectively. Interestingly, of the six transcripts encoded by this locus, only the three containing both the *ylqD* and *rimM* ORFs were downregulated upon RNase P depletion (Fig. 4B).

### A determinant for downregulation of the *rimM* operon is located within the *ylqD* ORF.

**FIG 5:**
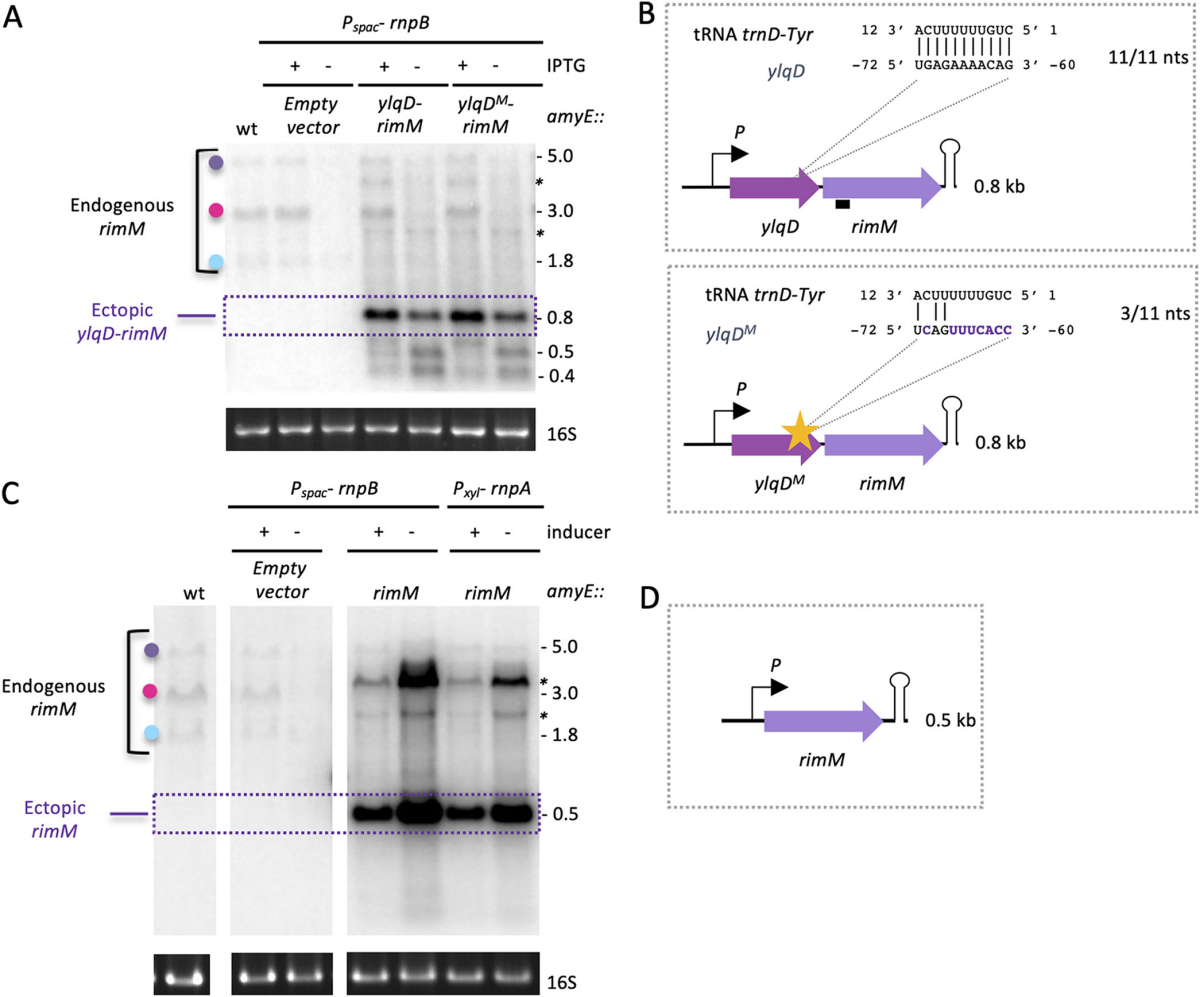
A determinant for downregulation of the *rimM* operon in response to tRNA maturase depletion is located within the *ylqD* ORF. (A) Northern blot of total RNA from *Pspac*-*rnpB* cells isolated in the presence or absence of IPTG showing the effect of RnpB depletion on expression of an ectopic *ylqD*-*rimM* short operon containing a WT or mutated (M) potential target sequence for *trnD*-Tyr pre-tRNA within the *ylqD* ORF. Colored dots identifying endogenous *rimM* transcripts follow the code used in Fig. 4. (B) Schematic of ectopic *ylqD*-*rimM* constructs placed under the control of the constitutive promoter (P) used for panel A. The enlargement shows the complementarity to the *trnD*-Tyr pre-tRNA and its disruption in the *ylqD*^M^-*rimM* mutant construct. Coordinates are relative to the start codon of *rimM*. The black box indicates the location of the *rimM* probe. (C) Northern blot showing the effect of RNase P depletion (*rnpA* or *rnpB*) on expression of an ectopic *rimM*-only construct. (D) Schematic of ectopic *rimM* construct placed under the control of the constitutive promoter (P) used for panel C. Slower-migrating bands (marked with asterisks) are likely due to read-through of the terminator in the ectopic construct. 16S rRNA levels (ethidium bromide stained) are shown as a loading control.

To further narrow down which ORF was responsible for downregulation of *rimM* operon expression, we subcloned the *ylqD*-*rimM* or *rimM*-only parts of the operon under the control of a *Pspac* promoter, rendered constitutive by deleting the *lac* operator [Pspac(con)] (Table S3), with an artificial terminator hairpin to provide a defined 3′ end. The constructs were integrated into the chromosome at the *amyE* locus, and levels of the ectopic transcript were analyzed by Northern blotting in RNase P-depleted cells using a probe specific for *rimM*. The steady-state levels of the synthetic *ylqD*-*rimM* transcript were downregulated in response to RNase P depletion, albeit not as dramatically as the native operon (1.3- versus 2.3-fold), suggesting that a determinant involved in downregulation is still included in this shorter construct (Fig. 5A and B). Two degradation intermediates (~0.5 and ~0.4 kb) of the *ylqD*-*rimM* transcript also accumulated, suggesting that this transcript is cleaved twice under conditions of RnpB depletion. It is possible that the weaker effect of RnpB depletion on the full-length transcript and the accumulation of visible degradation intermediates is explained by the presence of a stabilizing terminator hairpin at the 3′ end of each of these species that is not present in the native mRNA. Intriguingly, the construct containing only the *rimM* ORF (with the same 3′ terminator) was upregulated in response to RNase P depletion (Fig. 5C and D). In combination, these results suggest that the region responsible for posttranscriptional downregulation of *rimM*-containing transcripts upon depletion of RNase P is located primarily within the *ylqD* ORF.

Since unprocessed tRNAs accumulate in RNase P- and RNase Z-depleted cells, we wondered whether they could act as potential posttranscriptional regulators of target mRNAs by base pairing to their targets via their single-stranded 5′ and 3′ extensions. Using TargetRNA2 (12), a prediction program used for identifying targets of small RNAs (sRNAs) in bacteria, we identified an 11-nt region within the *ylqD* ORF that could potentially base pair with the 5′ immature extension of unprocessed *trnD*-Tyr tRNA (Fig. 5B). To test whether this sequence was involved in downregulation of the *ylqD*-*rimM* construct in cells depleted of RNase P, we weakened the putative base-pairing interaction by introducing mutations into the *ylqD* mRNA sequence (while maintaining the YlqD amino acid sequence as much as possible) (Fig. 5B). The mutant *ylqD*^M^-*rimM* construct was downregulated and processed similarly to the WT under conditions of RNase P depletion, suggesting that 5′-extended *trnD*-Tyr does not act as a posttranscriptional regulator of this operon. For the moment, the sequence element(s) within *ylqD* responsible for downregulation of the *rimM* operon under conditions of tRNA maturase depletion remains unknown.

### Downregulation of *rimM* expression under physiological conditions resulting in reduced *rnpA* expression is independent of immature-tRNA accumulation.

**FIG 6:**
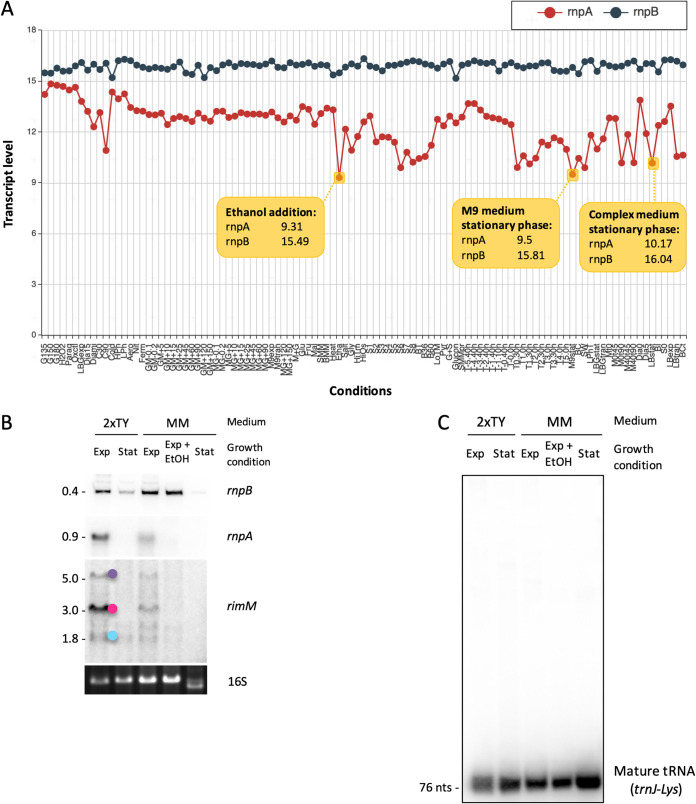
Ethanol stress and stationary phase affect *rnpA* and *rnpB* expression without causing tRNA processing defects. (A) *rnpB* (black) and *rnpA* (red) transcript levels over 100 different growth conditions (from reference 10), adapted from the *Subti*Wiki website (http://subtiwiki.uni-goettingen.de/). The three conditions indicated in yellow (ethanol stress and stationary phase in complex and minimal medium) result in reduced *rnpA* RNA levels. For each condition, *rnpA* and *rnpB* RNA levels (log_2_) are indicated in the yellow box. (B) Northern blot comparing *rnpB* (first panel; acrylamide gel because of small size, 401 nt), *rnpA* and *rimM* (second and third panels; agarose gel) RNA levels, after ethanol addition (EtOH) or during exponential (Exp) or stationary (Stat) phase in minimal (MM) or complex (2xTY) medium. Colored dots identifying *rimM* transcripts follow the code used in Fig. 4. Note that the 16S rRNA (loading control) is beginning to be degraded in MM in stationary phase. (C) Northern blot probed for *trnJ*-Lys (acrylamide gel) showing no pre-tRNA accumulation in the different conditions tested. See Figure S1 in reference 8 for accumulation of *trnJ*-Lys precursors under conditions of RnpA and RnpB depletion.

We next asked whether the downregulation of the *rimM operon* we observed during RNase P depletion would also occur under physiological conditions where RNase P expression is reduced. The level of expression of the *rnpB* RNA is relatively constant in tiling array experiments in over 100 conditions tested, whereas *rnpA* mRNA levels decrease upon ethanol addition and during stationary phase in both complex and minimal media (Fig. 6A) (10). We confirmed that *rnpA* RNA expression was reduced to levels below detection in these three conditions in comparison with exponential growth in the respective medium, by Northern blotting (Fig. 6B). Ethanol treatment did not affect *rnpB* RNA levels; however, they were reduced during stationary phase in both minimal and complex media, in contrast to the tiling array data. The expression of *rimM* varied similarly to that of *rnpA* under the conditions tested (Fig. 6B).

We next asked whether tRNA maturation was affected in stationary phase or upon addition of ethanol using a probe for *trnJ*-Lys tRNA. Surprisingly, despite the decreased levels of the *rnpA* mRNA in all three conditions, and of *rnpB* in stationary phase, we did not observe an accumulation of pre-tRNAs (Fig. 6C). It is possible that very few new tRNA molecules are synthesized under these conditions and/or that the remaining cellular RNase P activity provided by the stable RnpA protein and RnpB RNA is sufficient to ensure the processing of any that are transcribed. In either case, these experiments suggest that the downregulation of *rimM* expression that accompanies the decrease in *rnpA* and *rnpB* expression in stationary phase or during ethanol stress is more related to growth arrest than an accumulation of immature tRNAs.

### Downregulation of *rimM* in RNase P depletion strains depends partially on (p)ppGpp production.

**FIG 7:**
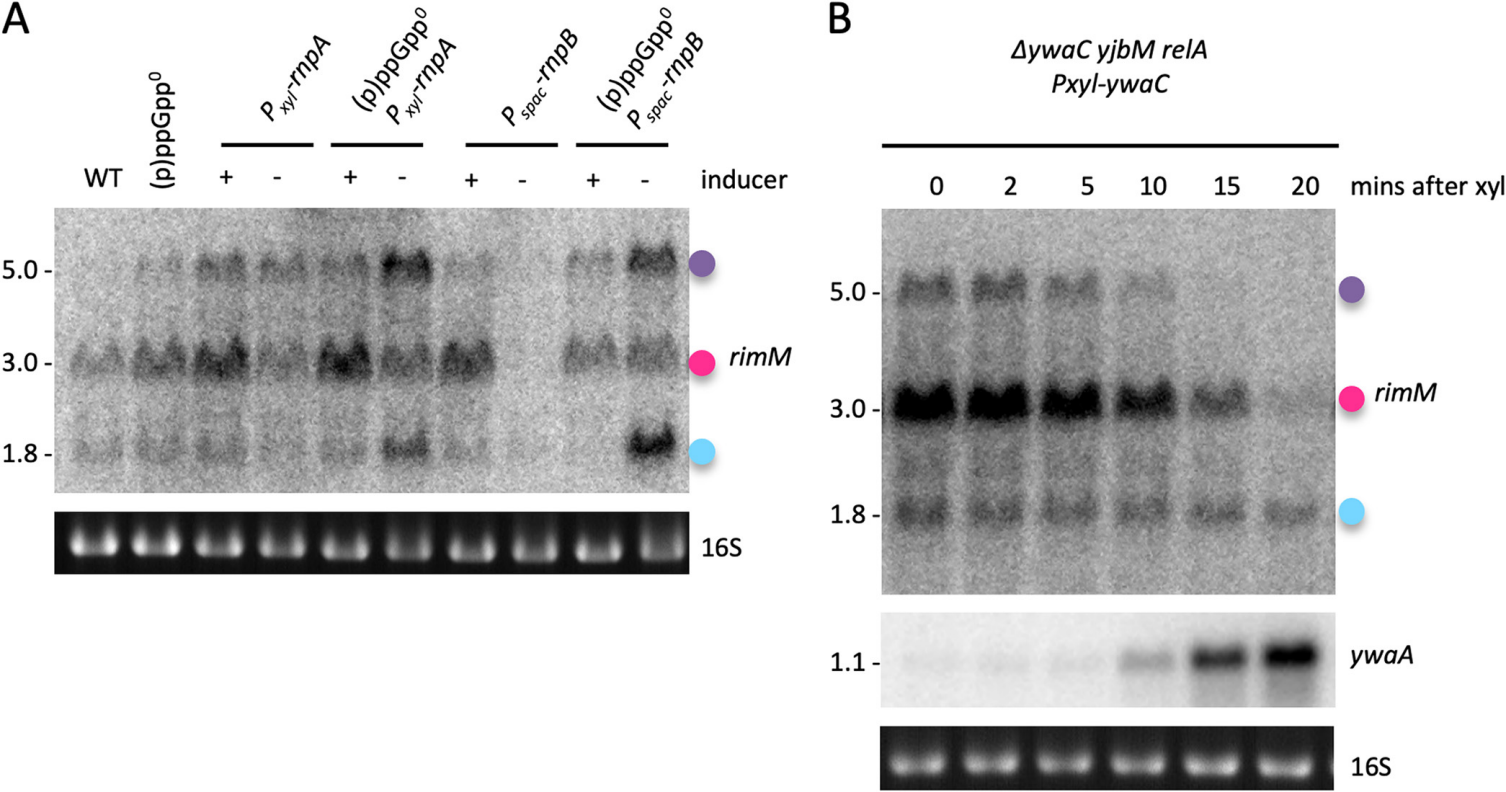
Influence of (p)ppGpp on *rimM* expression. (A) Northern blot comparing the effect of RNase P depletion (RnpB or RnpA) on *rimM* expression in a WT and (p)ppGpp^0^ background. (B) Northern blot of *rimM* expression after induction of (p)ppGpp production using a xylose-inducible *ywaC* gene in a Δ*yjbM ywaC relA* [(p)ppGpp^0^] background. Colored dots identifying *rimM* transcripts follow the code used in Fig. 4. 16S rRNA levels (ethidium bromide stained) are shown as a loading control. Series of blots where a single loading control is shown were stripped and reprobed. Note that this Northern blot was generated by reprobing a membrane previously used in reference 8, with permission to reuse the *ywaA* control panel granted by the publisher.

In bacteria, both stationary phase and ethanol stress are associated with increased production of (p)ppGpp, hyperphosphorylated guanosine derivatives that are known to globally reprogram transcription (13, 14). Considering that tRNA maturase-depleted cells also trigger a RelA-dependent production of (p)ppGpp (8), we asked whether *rimM* downregulation in these cells was dependent on (p)ppGpp production, by measuring *rimM* expression in (p)ppGpp^0^ strains depleted of RnpA or RnpB. The (p)ppGpp^0^ strain lacks the three genes encoding (p)ppGpp-synthesizing enzymes in B. subtilis (*yjbM*, *ywaC*, and *relA*) (15). If (p)ppGpp were the key mediator, we would expect the effect of RNase P depletion on *rimM* expression to be reduced or abolished in the (p)ppGpp^0^ background. Rather than simply abolishing the effect, *rimM* transcripts actually showed higher levels in the tRNA maturase-depleted (p)ppGpp^0^ strains compared to the RnpA- and RnpB-depleted strains capable of making (p)ppGpp (Fig. 7A), suggesting that (p)ppGpp has an independent repressive effect on *rimM* mRNA levels.

We thus assessed whether the alarmone (p)ppGpp could downregulate *rimM* expression in the absence of a tRNA processing defect using an engineered strain that allows us to produce (p)ppGpp in the absence of immature tRNA accumulation or nutrient starvation (8). This (p)ppGpp^+^ strain consists of an ectopic copy of the *ywaC* gene placed under the control of a *Pxyl* promoter in the (p)ppGpp^0^ strain background. We used derepression of the CodY-regulated *ywaA* mRNA as a proxy to follow the increase in (p)ppGpp levels in this strain *in vivo* (Fig. 7B) (8). We observed that (p)ppGpp induction alone had no effect on the small (Y/P_2_-T_2_) (Fig. 7B, cyan dot) *rimM* transcript, whereas the two larger species (Y/P_2_-T_3_ and P_1_-T_3_) (Fig. 7B, pink and purple dots, respectively) were downregulated as the expression of the (p)ppGpp reporter *ywaA* increased (Fig. 7B). Although (p)ppGpp production alone recapitulated what was seen in RnpB-depleted cells, the fact that only the two larger transcripts behaved as expected from the results obtained in the RNase P-depleted ppGpp^0^ strain (Fig. 7A) suggests that regulation of the smallest transcript (Y/P_2_-T_2_) is more complex than simple transcriptional repression by (p)ppGpp.

**FIG 8:**
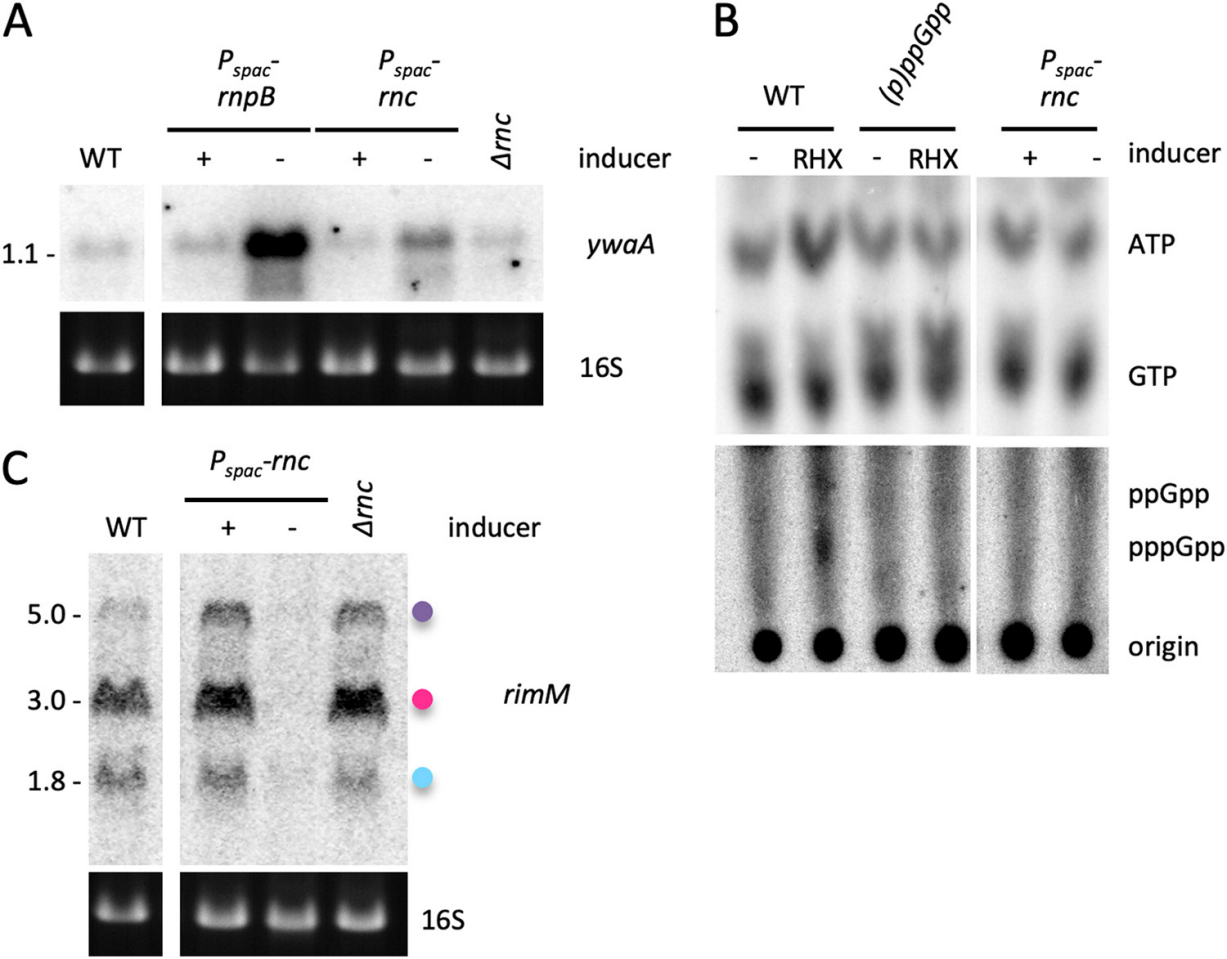
Expression of the *rimM* operon is regulated by growth rate independently of (p)ppGpp. (A) Northern blot comparing the effect of RNase III deletion and RNase P or RNase III depletion on derepression of the CodY-regulated *ywaA mRNA.* (B) RNase III-depleted cells do not accumulate large amounts of (p)ppGpp compared to tRNA maturase-depleted ones. TLC analysis of ^32^P-labeled nucleotides extracted from RNase III-depleted cells (*rnc*). Arginine hydroxamate (RHX; 250 mg/mL) was added to WT and (p)ppGpp^0^ strains as positive and negative controls. The top and bottom halves were exposed for different times. Note that this is a recrop of an image previously published in reference 8; the first four (control) lanes are reproduced to show the migration position of (p)ppGpp, with permission from the publisher. (C) Northern blot comparing the effect of RNase III depletion or deletion on *rimM* expression. Colored dots identifying *rimM* transcripts follow the code used in Fig. 4. 16S rRNA levels (ethidium bromide stained) are shown as a loading control.

The fact that the Y/P_2_-T_2_ *rimM* transcript could be downregulated independently of (p)ppGpp production led us to investigate the possibility of a further layer of regulation where the growth slowdown in tRNA maturase-depleted cells would also affect *rimM* expression by a mechanism independent of alarmone levels. To test this, we sought to reproduce the growth rate defect by depleting for an unrelated essential enzyme. We therefore performed Northern blot analysis on total RNA extracted from both RNase III (*rnc*) depletion and deletion strains. The double-strand-specific endoribonuclease RNase III is essential in B. subtilis because it is required to silence expression of foreign toxin genes of two prophages (*txpA* in the prophage-like element *skin* and *yonT* in prophage SPβ) (16). Whereas depletion of RNase III in a WT background leads to growth arrest, the *rnc* gene can be deleted in a strain lacking the two prophage toxins without a marked effect on growth rate. The depletion of RNase III in a toxin-WT background led to a very limited derepression of the CodY regulon in comparison with tRNA maturase-depleted strains (Fig. 8A) and did not result in accumulation of visible amounts of (p)ppGpp on thin-layer chromatography (TLC) (Fig. 8B). This validates the use of RNase III depletion strains to examine the effect of growth rate on *rimM* expression and to distinguish this from the effect of accumulating high levels of (p)ppGpp. While RNase III deletion (in the *ΔtxpA ΔyonT* background) had no effect on *rimM* expression, all three *rimM*-containing transcripts were strongly downregulated during RNase III depletion (in the toxin-WT background) (Fig. 8C), confirming that growth rate also plays a major role in the regulation of *rimM* expression, independently of (p)ppGpp and tRNA maturase depletion.

## DISCUSSION

This study began with the observation that depletion of tRNA maturase enzymes in B. subtilis led to a defect in 30S ribosome subunit assembly, which we showed was in part due to an accumulation of (p)ppGpp and an inhibition of the activity of 30S assembly GTPases (8). In our early attempts to understand the mechanism underlying this phenomenon, we studied the expression of several 30S assembly factors and discovered that most were either up- or downregulated at the mRNA level upon depletion of either RNase P or RNase Z.

We initially focused on the *rimM* mRNA because the 30S ribosome assembly defect observed in tRNA maturase depletion mutants was very similar to that seen in a *ΔrimM* strain. Although we later showed that ectopic *rimM* expression could not correct the assembly defect in the *rnpB*-depleted strain (8), we were nonetheless curious about how *rimM* expression was affected by the decrease in the levels of mature tRNAs. Together, our data (summarized in Fig. S4) indicate that the downregulation of *rimM* transcript levels in tRNA maturase-depleted cells is the result of a complex mixture of transcriptional and posttranscriptional mechanisms, caused by a combination of effects mediated by a reduction in growth rate, (p)ppGpp production, and a translational defect due to lack of functional tRNAs, with each layer of regulation capable of functioning independently of the others and affecting the three *rimM* transcripts distinctly at different levels of severity.

We hypothesized that the accumulation of immature tRNAs during tRNA maturase depletion increases ribosome stalling. Stalled ribosomes are known to affect mRNA decay in bacteria (17), and a tRNA loss-of-function mutation leading to pre-tRNA processing defects was reported to induce ribosome stalling in mice (9). In agreement with our hypothesis, we observed that treatment with the translation elongation inhibitor chloramphenicol at the MIC recapitulates the effects of tRNA maturase depletion on the mRNA levels of several different assembly factor mRNAs tested, four of which were upregulated (*era*, *yqeH*, *ydaF*, and *yjcK*) and two downregulated (*rimM* and *cpgA*). Interestingly, Cm treatment at subinhibitory concentrations did not impact cofactor mRNA levels in the same way, with low Cm concentrations initially having transitory up-effects that were then reversed at longer incubation times. One possibility is that short ribosome stalls transiently block access to cleavage sites by housekeeping RNases such as RNase Y, resulting in mRNA stabilization, while prolonged stalling could lead to mRNA destabilization by an enzyme such as Rae1, proposed to enter the A site of stalled ribosomes (2), or by leaving large stretches of mRNA unoccupied by ribosomes and vulnerable to cleavage by canonical degradation pathways. Another notable difference between the two conditions is that the stringent response is induced by Cm at the MIC, as evidenced by the increase in expression the *ilvA* mRNA from the CodY regulon (Fig. S5), which could also contribute to the increased severity of the response to higher Cm concentrations. Activation of the stringent response in Cm-treated B. subtilis was also previously observed (18), consistent with our results. This is a marked difference from E. coli, where Cm is a known inhibitor of stringent response induction (19, 20). The mechanism still remains elusive in both cases.

Beyond their canonical role in protein synthesis, tRNAs have been implicated in the regulation of several biological processes (for reviews, see references 21 and 22). A new class of small noncoding RNAs has emerged recently called tRNA-derived fragments (tRFs) or tRNA-derived small RNAs, whose biological roles are not yet well understood (23). Different types of tRFs differ in the cleavage position of the mature or precursor tRNA transcript. They have been particularly studied in humans, where they have been shown to be involved in regulation of a variety of cellular processes, including global translation, cellular proliferation, apoptosis, and epigenetic inheritance (24). Interestingly, a 3′ tRF in human cells plays an essential role in fine-tuning ribosome biogenesis under normal physiological conditions by posttranscriptionally regulating translation of at least two r-protein mRNAs (25). Although tRFs have not yet been identified in B. subtilis, we asked whether pre-tRNAs could bind certain assembly factor mRNAs via their 5′ or 3′ extensions and cause some of the posttranscriptional effects observed in the tRNA maturase depletion strains. tRFs with 5′ or 3′ extensions (pre-tRFs) could similarly behave as a new pool of potential regulatory sRNAs. Although the potential base-pairing we identified between the 5′ extension of *trnD*-Tyr and the *rimM* transcripts does not seem to play a role in the downregulation of *rimM* expression, this does not preclude the possibility that other pre-tRNAs or pre-tRFs could be involved in posttranscriptional regulatory events in B. subtilis.

In this study, we focused on the consequences of tRNA maturase depletion on ribogenesis cofactors mRNAs; however, the effects we uncovered are likely not restricted to this category of genes, as exemplified by the effects on the stabilities of the *yrzI* and *bmrCD* mRNAs. Along these lines, a recent study in E. coli showed that the abundance of 46% of transcripts were affected in a strain where the protein moiety of RNase P was heat denatured (26). The observations that the addition of chloramphenicol mimicked the effect of tRNA maturase depletion in B. subtilis for the upregulated mRNAs and that downregulation was the net result of a mixture of translational and transcriptional effects suggest that the effects seen on the E. coli transcriptome may substantially be the result of ribosome stalling on mRNAs due to lack of functional tRNAs, with differential impacts (up, down or neutral) on individual mRNA stabilities or transcription levels. More detailed studies are required to untangle these effects on a global level in both organisms.

## MATERIALS AND METHODS

### Strains and culture conditions.

All B. subtilis strains used were derived from our laboratory strain SSB1002, a W168 *trp^+^* prototrophic strain. Strains are listed in Table S1, and details of strains and plasmid constructs are provided in Table S2 and S3, respectively. Oligonucleotides used are listed in Table S4.

Unless stated otherwise, B. subtilis strains were grown in 2× YT liquid medium (1.6% peptone, 1% yeast extract, 1% NaCl) at 200 rpm at 37°C in ≤1/10 volume of the flask to ensure proper aeration. Overnight precultures were grown in the presence of appropriate antibiotics and inducer (1 mM IPTG or 2% xylose), in the case of depletion strains. Experimental cultures were grown in the absence of antibiotics, except where stated. For depletion strains, overnight induced cultures were washed three times with prewarmed 2× YT medium and inoculated at an optical density at 600 nm (OD_600_) between 0.02 and 0.2, depending on the strain, in fresh medium with or without inducer. Generally, induced cells were harvested for RNA preparation around an OD_600_ of 0.6, and cells grown in the absence of the inducer were followed until they reach a plateau before being harvested. Inoculation and depletion conditions were determined empirically for each strain, such that the depleted cells were harvested between OD_600_ of 0.3 and 0.7. For RnpA depletion, cultures were inoculated at an OD_600_ of 0.05 in the presence of 2% xylose (inducer) or 2% glucose to tighten repression of the *Pxyl* promoter, which typically led to a growth arrest (plateau) around an OD_600_ of 0.6. For *rnz* and *rnpB* depletion strains, cultures were inoculated in presence or in the absence of 1 mM IPTG at OD_600_ of 0.05 and 0.2, respectively. RNase Z and RnpB depleted cells typically plateau around OD_600_ of 0.6 and 0.3, respectively.

For rifampicin experiments, B. subtilis strains were grown in 2× YT at 37°C with shaking as described above. At an OD_600_ of 0.6 (or less for some depletion strains), rifampicin was added to a final concentration of 150 μg/mL in order to block new RNA synthesis. Samples were collected at different time points (e.g., 0, 2, 5, 10, 15, and 20 min) by mixing the cells with frozen 10 mM sodium azide (200 μL for 1.3 mL culture). Samples were vortexed until the sodium azide thawed, cells were pelleted by centrifugation at 4°C, and the pellet was conserved at −20°C until RNA extraction.

To mimic amino acid starvation, we depleted charged arginine tRNAs by addition of arginine hydroxamate (RHX) at 250 mg/mL in cultures growing in 2× YT at an OD_600_ of 0.3.

To study the effect of translation pausing, we added the translation elongation inhibitor Cm at a subinhibitory concentration (2.5 μg/mL) or the MIC (5 μg/mL) to cells growing in 2× YT at an OD_600_ of 0.6. Cells were harvested just before Cm addition (*t*_0_) and 15, 30, and 60 min after treatment.

To reproduce some growth conditions from the B. subtilis tiling array experiment (10) known to lead to a decrease in *rnpA* expression, ethanol was added to cultures growing in minimal medium (M9 with 0.5% glucose) at 4% (vol/vol) around an OD_600_ of 0.4, and cells were harvested 10 min after treatment.

### Plasmid constructs.

The *rimM* gene was amplified by PCR using the oligonucleotide pair CC2034/CC1986 and cloned between the BamHI and XhoI sites of the integrative pHM2-Pspac(con) vector (Table S3). The bicistronic *ylqD*-*rimM* construct was amplified by PCR using the oligonucleotide pair CC1985/CC1986 and cloned between the BamHI and SalI sites of pHM2-Pspac(con). The mutated construct *ylqD*^M^-*rimM* was obtained by two-fragment overlapping PCR. The upstream fragment was amplified with the forward primer CC1985 and the reverse primer CC2012, and the downstream fragment was amplified with the forward primer CC2011 and the reverse primer CC1986. The overlapping fragments were reamplified using the oligonucleotide pair CC1985/CC1986 and cloned between the BamHI and SalI sites of pHM2-Pspac(con). The integrative plasmids were linearized with XbaI before transformation, for integration into the *amyE* locus of the B. subtilis chromosome.

### RNA extraction and Northern blots.

RNA extraction was typically performed using the glass bead/phenol protocol (adapted from reference 27) on 1 to 8 mL mid-log-phase B. subtilis cells growing in 2× YT.

To perform Northern blotting, 5 μg total RNA was denatured for 5 min at 95°C in RNA gel loading dye (Thermo Scientific) before being separated on 1% agarose gels in 1× Tris-borate-EDTA (TBE) (native) or on denaturing 5% acrylamide gels in 1× TBE plus 7 M urea. RNA was transferred from agarose gels to a Hybond-N membrane (GE Healthcare) by capillary transfer for 4 h minimum in 1× transfer buffer (5× SSC [1× SSC is 0.15 M NaCl plus 0.015 M sodium citrate], 0.01 M NaOH). For Northern blots of acrylamide gels, RNA was electrotransferred at 4°C in 0.5× TBE for 4 h at 60 V or overnight at 12 V. RNA was cross-linked to the membrane by UV cross-linking at 120,000 μJ/cm^2^ using HL-200 Hybrilinker UV cross-linker (UVP). Probes for Northern blots were usually 25- to 30-nt DNA oligonucleotides radiolabeled on their 5′ ends by polynucleotide kinase. The *cpgA* mRNA was detected with a riboprobe by using a PCR fragment amplified using oligonucleotides CC2200 and CC2201 as the template. Membranes were preincubated in Ultra-Hyb (Life Technologies) for agarose blots or Roti-Hybri-Quick (Roth) for acrylamide blots for 1 h and hybridized with radiolabeled probes for a minimum of 4 h. Preincubation, hybridization, and wash steps were performed at 42°C in the case of 5′-labeled oligonucleotides or at 68°C for riboprobes. Membranes were quickly rinsed once at room temperature in 2× SSC–0.1% SDS to remove nonhybridized probe before being washed once for 5 min in the same buffer and then twice for 5 min in 0.2× SSC–0.1% SDS. Northern blots were exposed to PhosphorImager screens (GE Healthcare), and the signal was obtained by scanning with a Typhoon scanner (GE Healthcare) and analyzed by Fiji (ImageJ) software.

### TLC.

TLC analysis was used to detect radiolabeled (p)ppGpp as described in reference 8.

## References

[1] Condon C, Bechhofer DH. 2011. Regulated RNA stability in the Gram positives. Curr Opin Microbiol 14:148–154. doi:10.1016/j.mib.2011.01.010. 21334965 PMC3078962

[2] Leroy M, Piton J, Gilet L, Pellegrini O, Proux C, Coppee JY, Figaro S, Condon C. 2017. Rae1/YacP, a new endoribonuclease involved in ribosome-dependent mRNA decay in Bacillus subtilis. EMBO J 36:1167–1181. doi:10.15252/embj.201796540. 28363943 PMC5412759

[3] Condon C, Piton J, Braun F. 2018. Distribution of the ribosome associated endonuclease Rae1 and the potential role of conserved amino acids in codon recognition. RNA Biol 15:683–688. doi:10.1080/15476286.2018.1454250. 29557713 PMC6152456

[4] Braun F, Condon C. 2019. RNA processing, p 164–177. *In* Schmidt TM (ed), Encyclopedia of microbiology, 4th ed. Elsevier, Amsterdam, The Netherlands.

[5] Wen T, Oussenko IA, Pellegrini O, Bechhofer DH, Condon C. 2005. Ribonuclease PH plays a major role in the exonucleolytic maturation of CCA-containing tRNA precursors in Bacillus subtilis. Nucleic Acids Res 33:3636–3643. doi:10.1093/nar/gki675. 15983136 PMC1160522

[6] Pellegrini O, Li de la Sierra-Gallay I, Piton J, Gilet L, Condon C. 2012. Activation of tRNA maturation by downstream uracil residues in B. subtilis. Structure 20:1769–1777. doi:10.1016/j.str.2012.08.002. 22940585

[7] Marks J, Kannan K, Roncase EJ, Klepacki D, Kefi A, Orelle C, Vazquez-Laslop N, Mankin AS. 2016. Context-specific inhibition of translation by ribosomal antibiotics targeting the peptidyl transferase center. Proc Natl Acad Sci USA 113:12150–12155. doi:10.1073/pnas.1613055113. 27791002 PMC5086994

[8] Trinquier A, Ulmer JE, Gilet L, Figaro S, Hammann P, Kuhn L, Braun F, Condon C. 2019. tRNA, aturation defects lead to inhibition of rRNA processing via synthesis of pppGpp. Mol Cell 74:1227–1238.E3. doi:10.1016/j.molcel.2019.03.030. 31003868

[9] Ishimura R, Nagy G, Dotu I, Zhou H, Yang XL, Schimmel P, Senju S, Nishimura Y, Chuang JH, Ackerman SL. 2014. RNA function. Ribosome stalling induced by mutation of a CNS-specific tRNA causes neurodegeneration. Science 345:455–459. doi:10.1126/science.1249749. 25061210 PMC4281038

[10] Nicolas P, Mader U, Dervyn E, Rochat T, Leduc A, Pigeonneau N, Bidnenko E, Marchadier E, Hoebeke M, Aymerich S, Becher D, Bisicchia P, Botella E, Delumeau O, Doherty G, Denham EL, Fogg MJ, Fromion V, Goelzer A, Hansen A, Hartig E, Harwood CR, Homuth G, Jarmer H, Jules M, Klipp E, Le Chat L, Lecointe F, Lewis P, Liebermeister W, March A, Mars RA, Nannapaneni P, Noone D, Pohl S, Rinn B, Rugheimer F, Sappa PK, Samson F, Schaffer M, Schwikowski B, Steil L, Stulke J, Wiegert T, Devine KM, Wilkinson AJ, van Dijl JM, Hecker M, Volker U, Bessieres P, et al. 2012. Condition-dependent transcriptome reveals high-level regulatory architecture in Bacillus subtilis. Science 335:1103–1106. doi:10.1126/science.1206848. 22383849

[11] DeLoughery A, Lalanne JB, Losick R, Li GW. 2018. Maturation of polycistronic mRNAs by the endoribonuclease RNase Y and its associated Y-complex in Bacillus subtilis. Proc Natl Acad Sci USA 115:E5585–E5594. doi:10.1073/pnas.1803283115. 29794222 PMC6004469

[12] Kery MB, Feldman M, Livny J, Tjaden B. 2014. TargetRNA2: identifying targets of small regulatory RNAs in bacteria. Nucleic Acids Res 42:W124–W129. doi:10.1093/nar/gku317. 24753424 PMC4086111

[13] Murray HD, Schneider DA, Gourse RL. 2003. Control of rRNA expression by small molecules is dynamic and nonredundant. Mol Cell 12:125–134. doi:10.1016/s1097-2765(03)00266-1. 12887898

[14] VanBogelen RA, Kelley PM, Neidhardt FC. 1987. Differential induction of heat shock, SOS, and oxidation stress regulons and accumulation of nucleotides in Escherichia coli. J Bacteriol 169:26–32. doi:10.1128/jb.169.1.26-32.1987. 3539918 PMC211729

[15] Kriel A, Bittner AN, Kim SH, Liu K, Tehranchi AK, Zou WY, Rendon S, Chen R, Tu BP, Wang JD. 2012. Direct regulation of GTP homeostasis by (p)ppGpp: a critical component of viability and stress resistance. Mol Cell 48:231–241. doi:10.1016/j.molcel.2012.08.009. 22981860 PMC3483369

[16] Durand S, Gilet L, Condon C. 2012. The essential function of B. subtilis RNase III is to silence foreign toxin genes. PLoS Genet 8:e1003181. doi:10.1371/journal.pgen.1003181. 23300471 PMC3531473

[17] Deana A, Belasco JG. 2005. Lost in translation: the influence of ribosomes on bacterial mRNA decay. Genes Dev 19:2526–2533. doi:10.1101/gad.1348805. 16264189

[18] Rhaese HJ, Dichtelmuller H, Grade R. 1975. Studies on the control of development. Accumulation of guanosine tetraphosphate and pentaphosphate in response to inhibition of protein synthesis in *Bacillus subtilis*. Eur J Biochem 56:385–392. doi:10.1111/j.1432-1033.1975.tb02244.x. 809277

[19] Cashel M. 1969. The control of ribonucleic acid synthesis in Escherichia coli. IV. Relevance of unusual phosphorylated compounds from amino acid-starved stringent strains. J Biol Chem 244:3133–3141. doi:10.1016/S0021-9258(18)93106-6. 4893338

[20] Kurland CG, Maaloe O. 1962. Regulation of ribosomal and transfer RNA synthesis. J Mol Biol 4:193–210. doi:10.1016/s0022-2836(62)80051-5. 14460744

[21] Katz A, Elgamal S, Rajkovic A, Ibba M. 2016. Non-canonical roles of tRNAs and tRNA mimics in bacterial cell biology. Mol Microbiol 101:545–558. doi:10.1111/mmi.13419. 27169680 PMC5003029

[22] Raina M, Ibba M. 2014. tRNAs as regulators of biological processes. Front Genet 5:171. doi:10.3389/fgene.2014.00171. 24966867 PMC4052509

[23] Lee YS, Shibata Y, Malhotra A, Dutta A. 2009. A novel class of small RNAs: tRNA-derived RNA fragments (tRFs). Genes Dev 23:2639–2649. doi:10.1101/gad.1837609. 19933153 PMC2779758

[24] Kumar P, Kuscu C, Dutta A. 2016. Biogenesis and function of transfer RNA-related fragments (tRFs). Trends Biochem Sci 41:679–689. doi:10.1016/j.tibs.2016.05.004. 27263052 PMC5173347

[25] Kim HK, Fuchs G, Wang S, Wei W, Zhang Y, Park H, Roy-Chaudhuri B, Li P, Xu J, Chu K, Zhang F, Chua MS, So S, Zhang QC, Sarnow P, Kay MA. 2017. A transfer-RNA-derived small RNA regulates ribosome biogenesis. Nature 552:57–62. doi:10.1038/nature25005. 29186115 PMC6066594

[26] Mohanty BK, Kushner SR. 2022. Inactivation of RNase P in Escherichia coli significantly changes post-transcriptional RNA metabolism. Mol Microbiol 117:121–142. doi:10.1111/mmi.14808. 34486768 PMC8766891

[27] Bechhofer DH, Oussenko IA, Deikus G, Yao S, Mathy N, Condon C. 2008. Analysis of mRNA decay in Bacillus subtilis. Methods Enzymol 447:259–276. doi:10.1016/S0076-6879(08)02214-3. 19161848

