# Supplementary material for: Effect of tRNA Maturase Depletion on Levels and Stabilities of Ribosome Assembly Cofactor and Other mRNAs in Bacillus subtilis
Source: Microbiol Spectr. 2023 Feb 22;11(2):e05134-22. doi: 10.1128/spectrum.05134-22 (PMC10100781; doi:10.1128/spectrum.05134-22)
Supplement: Supplemental file 1 — Tables S1 to S4 and Fig. S1 to S5. Download spectrum.05134-22-s0001.pdf, PDF file, 2.6 MB [file spectrum.05134-22-s0001.pdf]

Table S1: *B. subtilis* strains used in this study

**Strains**	**Genotype**	**Reference**
SSB318	W168 *Pspac-rnpB:pMUTIN ery*	(1)
SSB1002	W168 *trp+*	Lab strain
CCB288	W168 *amyE::Pspac-rnc Cm rnc::spc pMAP65*	(2)
CCB321	W168 *Pspac-rnz:pMUTIN ery* pMAP65 *kan*	(3)
CCB418	W168 *txpA -10Δ yonT::ery rnc::spc*	(2)
CCB504	W168 *Pxyl-rnpA Cm*	(3)
CCB664	W168 *rimM*::*ery*	(4); This study
CCB994	W168 *Pspac-rnpB:pMUTIN ery amyE::pHM2-ylqD-rimM Cm*	This study
CCB1008	W168 *Pspac-rnpB:pMUTIN ery amyE::pHM2-*$ylqD^{M}$*-rimM Cm*	This study
CCB1014	W168 *Pspac-rnpB:pMUTIN ery amyE::pHM2 Cm*	This study
CCB1017	W168 *Pspac-rnpB:pMUTIN ery amyE::pHM2-rimM Cm*	This study
CCB1031	W168 *Pxyl-rnpA Cm amyE::pHM2-rimM Spc*	This study
CCB1050	W168 *yjbM::spc ywaC::kan relA::ery*	(3)
CCB1057	W168 *yjbM::spc ywaC::kan Pxyl-rnpA Cm relA::ery*	(3)
CCB1125	W168 *yjbM::spc ywaC::kan amyE::pX-ywaC Cm relA::ery*	(3)
CCB1137	W168 *yjbM::spc ywaC::kan Pspac-rnpB:pMUTIN relA::ery::tet*	(3)

Table S2. Details of strain construction

**Strain number**	**Plasmid**	**Description**	**Source/Ref.**
CCB994	752	Plasmid 752 linearized with XhoI and integrated into SSB318 *amyE* locus.	This study; (3)
CCB1008	762	Plasmid 762 linearized with XhoI and integrated into SSB318 *amyE* locus.	This study; (3)
CCB1014	606	Plasmid 606 linearized with XhoI and integrated into SSB318 *amyE* locus.	This study; (3)
CCB1017	764	Plasmid 764 linearized with SpeI and integrated into SSB318 *amyE* locus.	This study; (3)
CCB 1031	Plasmid 764 and pCS	First, plasmid 764 linearized with SpeI was integrated into *amyE* in WT. Then the plasmid 764 Cm cassette was switched to Spc using an antibiotic cassette switching vector (pCS). The resulting strain was then transformed with CCB504 gDNA.	This study; (3, 5)

Table S3: Plasmids constructed for this study

Plasmid number	Plasmid name	Initial vector	Insert	Oligos for PCR	Description	Source /Ref.
606	pHM2-Pspac(con)	pHM2	Pspac(con)		Promoter P_{spac} from pMUTIN-4M cloned without the operator sequence using EcoRI-HindIII restriction sites	This study; (6)
752	pHM2-Pspac(con)-ylqD-rimM	606	*ylqD-rimM*	CC1985 + CC1986	Insert amplified from gDNA and cloned in BamHI/SalI under plasmid 606 constitutive promoter.	This study
762	pHM2-Pspac(con)-ylqD^{M}-rimM	606	*ylqD^{M}-rimM*	CC1985 + CC2012; CC2011 + CC1986	ylqD^{M} and *rimM* were amplified from plasmid 752. The CC2011/CC2012 oligos introduce mutations in *ylqD* ORF. *ylqD^{M}-rimM* overlap was obtained by overlap PCR with underlined oligos and cloned in BamHI/SalI.	This study
764	pHM2-Pspac(con)-rimM	606	*rimM*	CC1986 + CC2034	Insert amplified from gDNA and cloned in BamHI/XhoI under plasmid 606 constitutive promoter.	This study

Table S4: Oligonucleotides used in this study

Oligo	Gene	Sequence
CC1005	*rnpA*	ACTGGCGGTTTGCAACTGATGTCCC
CC1006	*rnpB*	TGCGAGCATGGACTTTCCTCTACAG
CC1560	*rpsU*	CTAGCAGCTTCAGACTTTTTCTTGCGC
CC1841	*ylxS*	GAGTTCCAGCTGAAGGCTGTCTACGATTGGC
CC1842	*rbfA*	GCTTTTGCCAGCCCTTTCAGCGCTTCTTCCC
CC1845	*rimM*	GAAATCACCCGCACTTCGCCTTTGATTCCGTG
CC1846	*era*	CCTTGTTTCTCGTCGTTTGGGGCTTATCGC
CC1847	*yqeH*	CGACCAGAGAGTCCGTTTCTCCAATACCGTG
CC1915	trnJ-lys	GACTCGAACCTTCGACCCTCTGATTAAAAG
CC1985	ylqD fw	TAAGGATCCGCTTAGGCAAAATAGAAAAGCCTGGACGAG
CC1986	rimM rvs	TAACTCGAGAAAAAGGCCATCCGTCAGGATGGCCGAGCACGCCTTCAAACATTTaGGGAAACAGCG
CC2011	ylqD^{M} fw	GGGGATGACTGGCATcAGtttcaccCAGCGAACACCATCGTC
CC2012	ylqD^{M} rvs	GACGATGGTGTTCGCTGggtgaaaCTgATGCCAGTCATCCCC
CC2034	rimM fw	ATATGGATCCTGGAAGAGGTGATCATATGACAAAGCGATGG
CC2099	*ylqD*	CTCGGTTAAGACTTGCATAACGGCTACACGG
CC2100	*ylqC*	GTCATCTGGATGATCAACAAGCGGCGTCAC
CC2101	*trmD*	CTGCCTTTGATGTCAGGTCCTCGACCGCGTC
CC2103	*ffh*	GAAATCGTCTGCTGCAGTCGGTCGGCTAATC
CC2143	*rpsP*	GATGAAACGGCCGTCACGTGGTGAACGAGAATC
CC2144	*rplS*	CAGGACGGAACGCAGGAAGATCAGTACGAAG
CC2197	*yjcK*	CGAAACCGTCCCGATTAATCTATCGTCTGACG
CC2198	*ydaF*	GATTGTTTCTCGGTACGTGTCAGCGCTGCTTG
CC2199	*yfmL*	GACGAGCTGATCCGTTTCATCAAGCACGATC
CC2200	*cpgA fwd*	CAACGAGCTTATCAGGCCGCCAATTTGCAAC
CC2201	*cpgA + PT7 rvs*	gctctaatacgactcactatagggACGTGTGAATCAGCTCCACGTGGCGGG
CC2213	*ywaA*	GATGCAGAGGCGGTCGTTTGATTGATTCAG
CC2215	*ilvA*	CACATCCGGATCATCGAACGGATGGATAAACGTC
CC2450	*ksgA*	CTGTTTTCTCCGTCACTTCCGCGTGATCAAC

References to supplementary tables :

1. **Wegscheid B, Condon C, Hartmann RK.** 2006. Type A and B RNase P RNAs are interchangeable in vivo despite substantial biophysical differences. EMBO Rep 7:411-7.
2. **Durand S, Gilet L, Condon C.** 2012. The essential function of B. subtilis RNase III is to silence foreign toxin genes. PLoS Genet 8:e1003181.
3. **Trinquier A, Ulmer JE, Gilet L, Figaro S, Hammann P, Kuhn L, Braun F, Condon C.** 2019. tRNA Maturation Defects Lead to Inhibition of rRNA Processing via Synthesis of pppGpp. Mol Cell 74:1227-1238 e3.
4. **Koo BM, Kritikos G, Farelli JD, Todor H, Tong K, Kimsey H, Wapinski I, Galardini M, Cabal A, Peters JM, Hachmann AB, Rudner DZ, Allen KN, Typas A, Gross CA.** 2017. Construction and Analysis of Two Genome-Scale Deletion Libraries for Bacillus subtilis. Cell Syst 4:291-305 e7.
5. **Chary VK, Amaya EI, Piggot PJ.** 1997. Neomycin- and spectinomycin-resistance replacement vectors for Bacillus subtilis. FEMS Microbiol Lett 153:135-9.
6. **Gendron N, Putzer H, Grunberg-Manago M.** 1994. Expression of both *Bacillus subtilis* threonyl-tRNA synthetase genes is autogenously regulated. J Bacteriol 176:486-494.

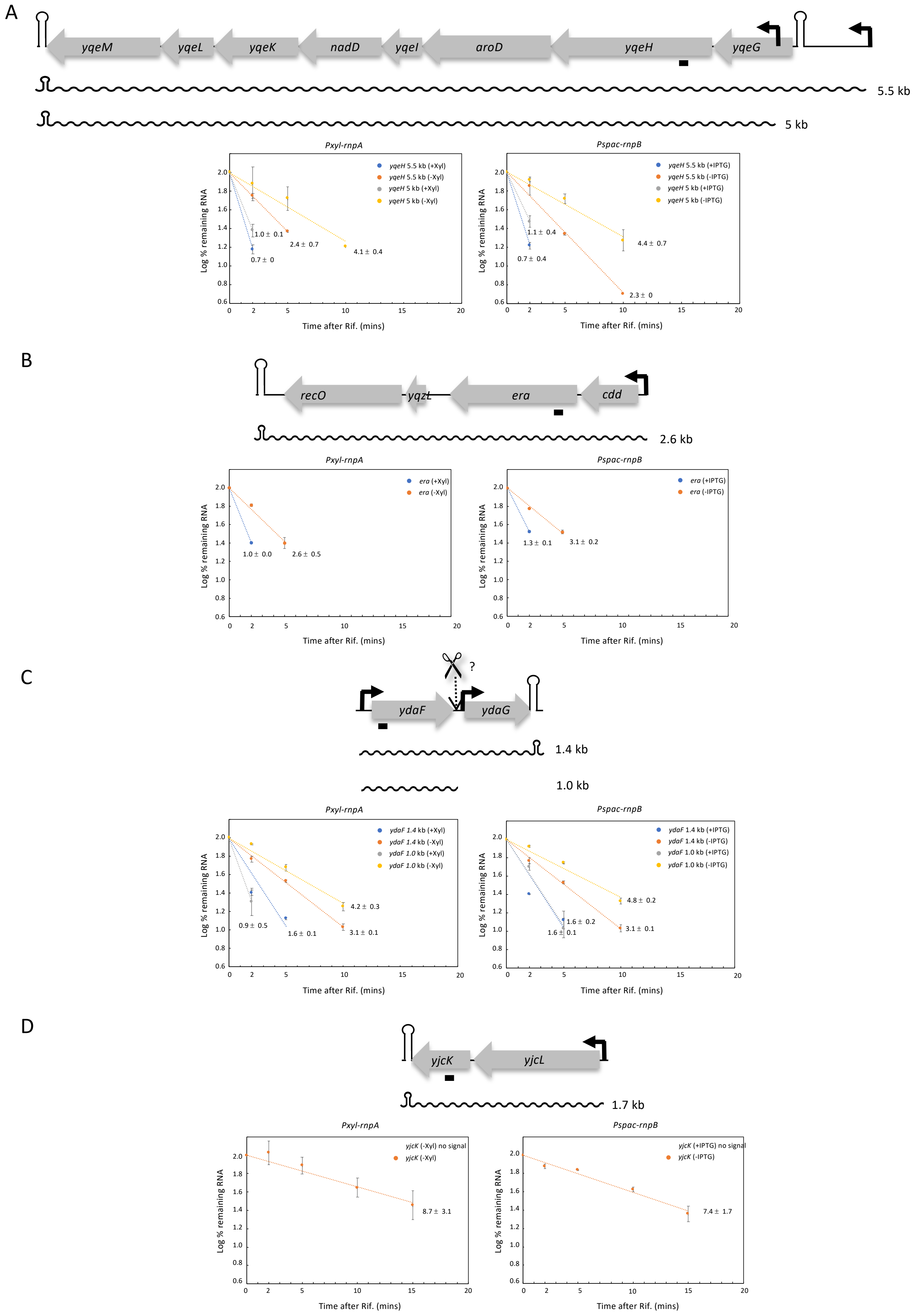

Figure S1: Schematics and RNA decay plots of up-regulated transcripts in RnpA and RnpB-depletion strains. Open reading frames (ORFs; not to scale) are shown as gray arrows and transcripts as wavy lines. Sizes are as indicated. Promoters are represented by black arrows and terminators as hairpins. Suspected endoribonucleolytic cleavage sites are indicated by a scissors symbol with a question mark. Quantifications are from the average of two experiments, with half-lives as indicated.

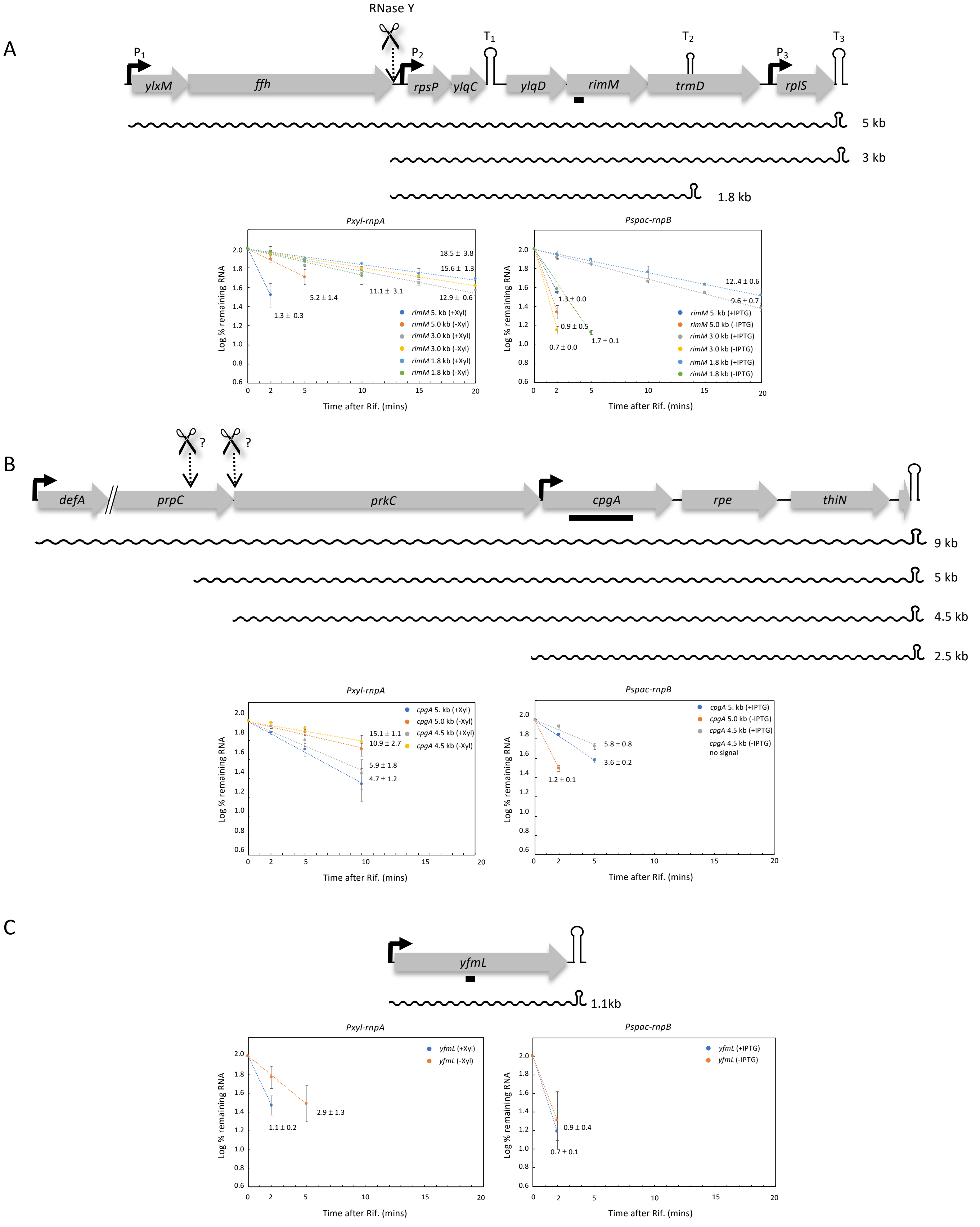

Figure S2: Schematics and RNA decay plots of down-regulated transcripts in RnpA and RnpB-depletion strains. Open reading frames (ORFs; not to scale) are shown as gray arrows and transcripts as wavy lines. Sizes are as indicated. Promoters are represented by black arrows and terminators as hairpins. Suspected endoribonucleolytic cleavage sites are indicated by scissors symbols with question marks. Quantifications are from the average of two experiments, with half-lives as indicated.

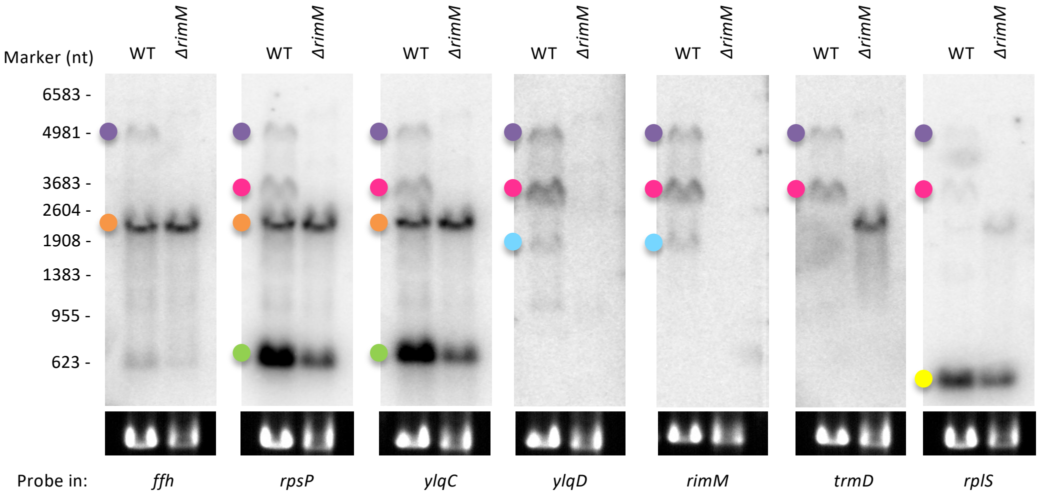

Figure S3: Northern blot analysis to determine the structure of the *rimM* operon. Total RNA from WT or *ΔrimM* strains was probed with oligonucleotides targeting different ORFs of the *rimM* operon (indicated below each panel). 16S rRNA levels (ethidium bromide stained) are shown as a loading control. The *ffh* and *ylqC* blots were stripped and reprobed for *rplS* and *ylqD*, respectively. Colored dots correspond to the color of transcripts shown in Figure 4A.

	levels			stability		Cm		ppGpp°		ppGpp$^+$	growth rate
	rnz	*rnpA*	*rnpB*	*rnpA*	*rnpB*	sub	MIC	*rnpA*	*rnpB*	-	*rnc*
FL	-	-	↓	↑	-	↷	↷	↑	↑	↓	↓
Y/P_2-T_3	↓	↓	↓	-	↓	↷	↷	-	↑	↓	↓
Y/P_2-T_2	↓	↓	-	-	↓	↷	↷	↑	↑	-	↓

Figure S4. Summary of effects on 3 three *rimM* containing transcripts. Data are taken from Northern blots shown in Figures 1 (levels), 2-3 (stability, Cm), 6A (ppGpp^0), 6B (ppGpp$^+$) and 7 (growth rate). Depleted tRNA maturases are indicated. Transcripts are listed according to size: full-length (FL), RNase Y/P_2-T_3 and RNase Y/P_2-T_2. Red arrows show down-effects, green arrows show up-effects, (-) no effect. Curved arrow in sub-inhibitory Cm condition (sub) reflects initial increase followed by decrease.

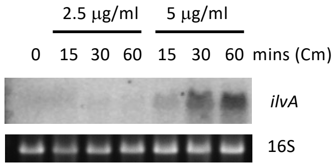

Figure S5. Induction of expression of the CodY regulon member *ilvA* by chloramphenicol. Northern blots of total RNA isolated at different times after addition of 0.5x MIC and MIC of chloramphenicol (Cm). 16S rRNA levels (ethidium bromide stained) are shown as a loading control.
